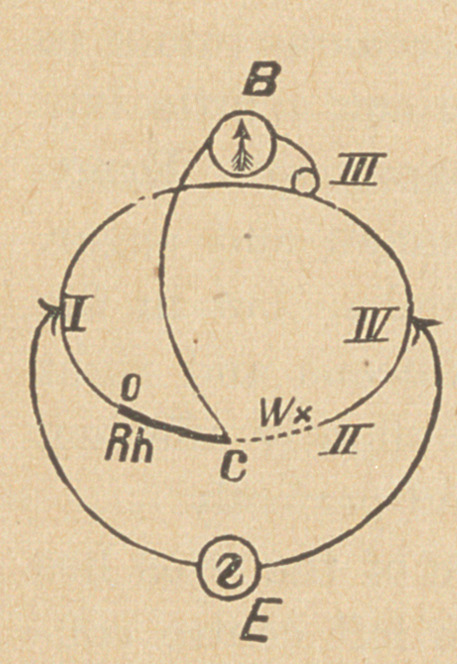# Electrical Phenomena in the Mouth

**Published:** 1883-02

**Authors:** Willoughby D. Miller

**Affiliations:** Berlin


### ELECTRICAL PHENOMENA IN THE MOUTH.

BY DR. WILLOUGHBY D. MILLER, OF BERLIN.

Quite a number of communications have lately been published on electrical phenomena in the mouth, as well as on the deleterious changes produced by them on filled and sound teeth, which are greatly in need of further elucidation. To solve the questions put forth in this connection, we need rational and exact observations and experiments, and cannot be satisfied, as heretofore, through striving to arrive at the desired information by means of *a priori* assumptions and theories.

To begin with the question whether, under physiological circumstances, any electric action can exist between the various portions of the living tooth itself, *i.e.*, between the enamel and the dentine, we must accept it as a strange fact that this very question seems to have attracted but little attention among the champions of the electric theory of caries.

If such an action did exist, it would be capable of destroying the teeth by local currents, and would afford an explanation of the origin of caries. But nothing of this kind has hitherto been observed, and we are not able to see at present how this question could be solved experimentally.

Consequently, another question comes to the front. What happens in electric phenomena when we connect a living tooth with a metallic filling? It has been proclaimed that "every tooth filled with metal forms a galvanic battery, which acts as soon as the surrounding fluids have an acid reaction."

But this supposition has not been verified by experimental facts. Even some of its champions have publicly acknowledged that, up to the present, nobody has been able to detect this galvanic action, much less to measure it.

Notwithstanding this, I claim that we can accept it as truth that electric currents do exist in the cavity of the mouth as soon as it contains teeth filled with metal. But these currents do not exist in such a manner, between the filling and the substance of the tooth, that the latter could be compared with one of the cells of a galvanic battery, but the electric phenomena which really take place in the mouth owe their existence to the dissimilarity of the nature of the substance of the metallic filling.

On the outside of every filling, even one of pure gold, electric currents will be generated if the filling is not of the same density throughout—a condition which will always exist in practice. These currents will flow between the dense and the less dense portions. But as by no means can it be said that all currents flow toward the edge of the filling, no harm can result to the tooth.

When two fillings of different materials occupy the same tooth, or come in contact in teeth which stand beside each other, a current is generated, flowing from the more easily oxydized metal (electro-positive) through the fluids of the mouth and teeth toward the less oxydizable metal (electro-negative), and which, while exercising a certain electric activity on the fluids of the oral cavity and on the tissues, is able to produce deleterious sequences. The current may liberate acids on the surface of the electro-positive metal, which can attack the tooth at the edge of the filling. But the action seems to stop as soon as the surface of the positive pole has been oxydized; in short, we have not yet found in practice that this process does any harm to the teeth.

When clasps of a baser metal are connected with a gold plate, after its insertion a weak current will be generated in the mouth. It may liberate acids about the clasps, and these acids, coming in contact with a tooth, might cause considerable erosion in the course of time. This action can be explained by the following experiment: I connected a bicuspid, by means of a platinum wire wound around its neck, with the positive pole of a Siemens battery of three cells, and another bicuspid in a similar manner to the negative pole of the same battery.

The teeth were immersed in a U-shaped tube, to a depth of two centimetres with a 0.75 per cent. solution of chloride of sodium.* The circuit contained for observation a galvanometer, whose multiplicator of 1,000 turns possessed a power of 200 Siemens ohms. On the closing of the circuit occurred a variation of 31 degrees; after being closed for two hours, this variation had decreased 8 degrees. When

*A solution of common salt can best be substituted for the animal fluids. It is indifferent to the organic tissues and has been tested in the experiments on muscle and nerve tissues in the laboratories, under the name of "physiological salt solution."

the teeth were taken from the fluid the positive tooth presented a furrow around it, at the place where the wire had surrounded it, of ½ mm. in depth, proving conclusively that the substance of the tooth had been destroyed by the acids liberated at the positive pole; on the negative tooth no change could be detected.

Consequently, electric currents can be generated in the mouth:

1. When a metal filling does not possess the same density throughout.

2. When two fillings of different metals come in contact.

3. When a plate or filling is composed of several different kinds of metal.

Another question in this connection concerns the conductivity of the tooth substance for electric currents.

It is well known that the different ingredients which compose the dentine are in themselves non-conductors of electricity, but I experimented to prove the dead-tooth substance a non-conductor in the following manner. A section of dentine of 0.03 mm. thickness was enclosed in a circuit composed of three Siemens cells, and the multiplicator rolls of a mirror galvanometer of 16,000 turns, with a resistance of 5,000 Siemens ohms. After the circuit was closed, I could not detect the slightest variation of the magnet mirror. This section of dentine was inclosed between the ends of two wires of 1.9 mm. diameter, and subjected to a pressure of about 3 grammes per square mm.

This experiment was varied, and three similar pieces were inclosed in the circuit, in such a manner that the connecting surfaces between the ends of the wire and the substance of the tooth were enlarged threefold. The variation always remained stationary, at zero: *i.e.*, the resistance was infinitely great.

If we connect the one pole of an electric battery of four Siemens' cells with the metallic filling of a tooth, and the other pole to a second filling, or to the gum tissue, we can plainly see that the circuit is closed. This fact has given rise to the false theory that dentine is a good conductor; but the current to be detected in this way is caused only by the fluids which are contained in the dental tubuli, the pulp-cavity and the root.

In the same manner the porous cylinder of a galvanic cell, in itself a non-conductor, becomes a good conductor as soon as moistened with the battery solution.

If, as thus claimed, the ability to conduct an electric current was dependent only on the fluids contained in the pores of the living tooth, it follows that a section of dead dentine moistened with a certain fluid would offer less resistance than a section of enamel moistened with the same, as dentine is more porous than enamel; further, a section of dentine crossing the dental tubuli at right angles would offer less resistance than one that runs parallel to them; and, lastly, the resistance would increase with the resistance of the fluid used for moistening the section.

These propositions were fully demonstrated by the following experiment:

The cross-section whose resistance was to be determined was held between the ends of two amalgamated zinc wires of 1.9 mm. diameter, pressed together by an even pressure of about 2 grammes to the square mm. The cross-section of the tooth, of a thickness of 0.04 mm., was moistened in its centre with concentrated zinc sulphate.

The arrangement of the apparatus of the Wheatstone bridge combination is as seen in the figure.

*E.* Two Siemens' closed cells.

*B.* A mirror galvanometer, after Wiedeman, of 16,000 turns, and a resistance of 5,000 Siemens' ohms, with a periodized magnet ring, after E. Du Bois-Raimond.*

*Rh.* The Rheostat.

*W.* The resistance to be measured.

0, *I.*, *II.*, *III.*, *IV.*, Connections with a round compensator, after Du Bois-Raimond,† with the modifications of Christiani.‡

A long series of experiments gave on an average, at the beginning, a resistance of 1,700 Siemens' ohms. for a section cut at right angles to the dental tubuli, and having a thickness of 0.04 mm. For another, of equal size, cut parallel, or nearly so, to the dental tubuli, the resistance was, in the beginning, about 8,050 Siemens' ohms. With progressing dryness, these resistances very quickly increased indefinitely.

When the same sections were moistened with water, a much

*Monthly report of the Royal Academy of Science at Berlin, 1869; p. 807, ff.

†Du Bois-Raimond's "Archives de la Physiologie," 1871; p. 608.

‡Poggdff. Annal. Erganzbd. VII; S. 573; Anmkg.

stronger resistance was found, which was, however, not measured, on account of the strong polarization.

That the resistanee of the enamel is much greater than that of the dentine, can be demonstrated by the following experiment:

Connect one end of a battery of four Siemens' cells with the gum, and the other end with the enamel of a tooth, and not the slightest sensation of an electric current will be perceptible; but if the second end is connected to the dentine, or to a metallic filling in contact with the dentine, a very disagreeable sensation will be perceptible, even if the battery be composed of but one element.

The experiments described herein have been made under the guidance of Prof. Christiani, in the laboratory of Prof. E. Du Bois-Raimond, and I take great pleasure in expressing my obligations to those two gentlemen.

## Figures and Tables

**Figure f1:**